# Unpriming or Strategizing?: A Critique of Sparrow and Wegner

**DOI:** 10.1371/journal.pone.0087512

**Published:** 2014-02-05

**Authors:** Miron Zuckerman, Jordan Silberman, Hoang Pham, Ista Zahn

**Affiliations:** 1 Department of Clinical and Social Sciences in Psychology, University of Rochester, Rochester, New York, United States of America; 2 Warner School of Education, University of Rochester, Rochester, New York, United States of America; 3 The Institute of Quantitative Social Science, Harvard University, Cambridge, Massachusetts, United States of America; University of Westminster, United Kingdom

## Abstract

When asked to randomly select answer choices on easy multiple choice questions, people select more correct answers than expected by chance. Sparrow and Wegner showed that this tendency was eliminated if participants answered questions correctly before answering randomly. They argued that answering a question correctly unprimes the tendency to choose the correct answer, thereby reducing the correct response rate close to the chance level of.5. An alternative explanation, consistent with these results, is that answering questions correctly provides a baseline, which allows participants to strategize, i.e., to match and mismatch equal numbers of their purportedly random responses to the baseline response. Three studies showed that the presence of a baseline, even when unpriming is not feasible, led to lower correct response rates than those obtained in a condition in which no baseline was available. Furthermore, the presence of a baseline led to more nonrandom sequences of correct and incorrect responses. One specific sequence–alternating correct and incorrect answers–mediated the relation between the presence of a baseline and lower correct response rate. These findings suggest that strategizing, not unpriming, accounts for Sparrow and Wegner’s results.

## Introduction

It is well established that the activation or priming of constructs linked to goal representation elicits goal-directed behavior. It also has been shown that primed behavior can be viewed as motivated behavior in that it is characterized by persistence and monitoring progress toward a goal [2], [3]. This priming-to-behavior process suggests that human behavior is determined to some extent by the stimuli that people encounter in their daily lives [4]. As pointed out by Sparrow and Wegner [1], however, there is a need to determine what unprimes behavior–what brings the priming process to a close. These authors proposed that allowing primed thoughts to occur leads to deactivation of the prime. They conducted a series of studies suggesting that, in their view, enactment of primes results in unpriming. The purpose of the present studies was to test a different behavioral model that may account for their results.

Sparrow and Wegner [1] examined unpriming in the context of the random answering paradigm [5]. In this paradigm, participants are asked to answer randomly a number of simple yes-no questions. Although random responding should result in an average of approximately 50% correct responses, participants answer questions correctly at significantly higher rates. Sparrow and Wegner suggested that the questions activate knowledge of the correct answers, which then influences or primes the actual answers. Sparrow and Wegner [1; Experiment 1] tested whether enactment of the primed behavior–i.e., answering the questions correctly–would result in unpriming. Participants were presented with a number of easy yes-no questions. In one condition (random-only), they were asked to answer randomly; in a second condition (correct-random), they were asked to answer each question correctly before answering it randomly. As predicted, the proportion of questions answered correctly in the correct-random condition (.49) was lower than that of the random-only condition (.58), and not higher than the level expected by chance. Sparrow and Wegner [1] concluded that “…responding (correctly) to a question may deactivate knowledge of the answer, allowing the respondent to answer the questions randomly later on” (p. 1012). However, Sparrow and Wegner did not test whether the answers in the correct-random condition were actually more random than those in the random-only condition.

We propose that answering the questions correctly before answering them randomly may facilitate a strategy that lowers the correct response rate but increases the non-randomness of responses. Correct responses may serve as a baseline such that when asked to answer randomly, participants can provide responses identical to the baseline about 50% of the time, and responses that are different from the baseline about 50% of the time. The easiest manner of doing so is to alternate between matching and mismatching baseline correct responses. Higher levels of alternating between correct and incorrect responses yield more runs (i.e., unbroken sequences of correct or incorrect answers) and, in particular, more one-value runs (i.e., correct, incorrect, correct, etc). More runs translate to a higher probability of alternation than the.5 probability that is expected in a random sequence. Because number of runs and the probability of alternation, p(A), are related {*p*(A) = (*r*−1)/(*n*−1), where *r* is the number of runs and *n* is the number of trials in a given sequence}, either the number of runs or *p*(A) can be used to evaluate whether a sequence is random [6]. Note that our prediction of lower randomness (as the result of alternating between correct and incorrect responses) is the exact opposite of Sparrow and Wegner’s [1] prediction of higher randomness (as the result of unpriming).

To test the strategizing interpretation, we replicated Sparrow and Wegner’s [1] Experiment 1 and added an incorrect-random condition in which participants were asked to answer each question incorrectly before answering it randomly. If correct-random instructions allow participants to form a baseline, so should incorrect-random instructions. Specifically, participants can match and mismatch their purportedly random responses to incorrect baseline responses as much as they can match and mismatch the random responses to correct baseline responses. It was predicted, therefore, that both the correct-random and the incorrect-random conditions (in comparison to the random-only condition) would lower the correct response rates. Furthermore, we predicted that both the correct-random and the incorrect-random conditions would yield less random response patterns, which would mediate the effect of experimental condition on level of correct responses.

One might argue that generating incorrect responses in the incorrect-random condition may cause participants to think about the correct responses (before identifying the incorrect response), which in turn may lead to unpriming in the same way that correct responses do. According to this argument, unpriming can account for lower correct response rate in both the correct-random and the incorrect-random conditions. Our prediction, however, is that the lower correct response rate in these two conditions is mediated by less random response patterns and, more specifically, by alternating between correct and incorrect responses. Unpriming, according to Sparrow and Wegner [1], would be expected to generate more random response patterns, which in turn would lead to lower correct response rates (see quote above). These two contradictory predictions were examined in Experiment 1 (we should also note that proposing that thinking about correct answers but responding incorrectly generates unpriming also raises a logical problem and is not supported empirically–issues that will be addressed in the general discussion).

## Experiment 1

Participants were assigned to one of three conditions: correct-random, incorrect-random, and random-only. In the correct-random and incorrect random conditions, participants were asked to answer each question correctly or incorrectly, respectively, before answering it randomly. In the random-only condition, participants were asked only to answer randomly. The instructions followed, verbatim when possible, those of Sparrow and Wegner [1; Experiment 1, p. 1012). Importantly, the request to generate random responses asked that participants not use any strategy such as generating yes/no/yes/no sequences but, instead, “flip a coin in your head” to determine the answer. Nothing was mentioned about the expected correct response rate.

### Methods

#### Participants

Seventy-five undergraduates (53 females) took part in the study in exchange for course credit. This study and the two that follow were approved by the University of Rochester Institutional Review Board. Participants in all three studies signed consent to procedure forms prior to the study and consent to use of their data forms after debriefing.

#### Procedure

The entire procedure was administered by a computer, using MediaLab 2006.2 [7]. Using a pair of keys labeled yes and no, participants answered 60 easy questions (e.g., “Does a triangle have three sides?”). The correct answer was “yes” for half of the questions but this information and the number of questions were not disclosed to participants. The order of whether “yes” or “no” served as the correct response was random. For participants in the random-only condition, each question appeared with the instruction to answer randomly. For participants in the correct-random and incorrect-random conditions, each questions appeared twice–once with instructions to answer correctly or incorrectly, and a second time with instructions to answer randomly. Inter-trial intervals were 2 seconds long.

### Results and Discussion

**Figure 1:**
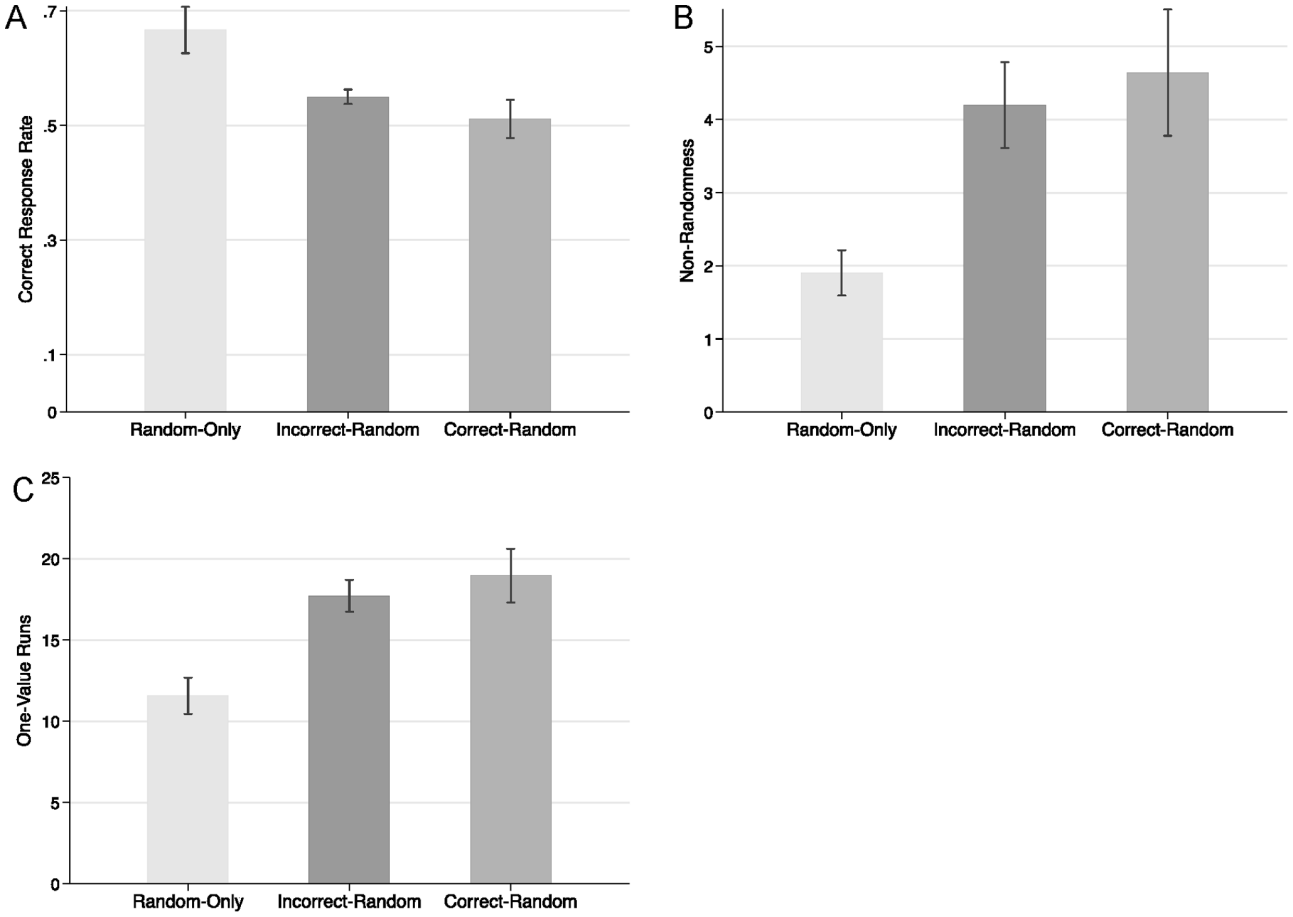
Means for (A) correct response rate, (B) non-randomness, and (C) one-value runs by experimental condition (Experiment 1). Error bars are standard errors of the means.

Gender did not have any significant effects in this and the following studies, ps >.10, and was thus removed from all analyses. Participants in the random-only condition had a higher correct response rate (M = .67) than participants in the correct-random (M = .51) and incorrect-random (M = .55) conditions (see Figure 1a). Table 1, top line, presents the relevant contrasts. Both the correct-random and the incorrect-random conditions differed significantly from the random-only condition in correct response rates.

**Table 1:** t-tests of Contrasts Among Conditions (Experiment 1).

Type of Contrast				
Dependent variable	Correct-random vs. random-only		Incorrect-random vs. random-only	
	t	r	t	r
Proportion of correct responses	3.55***	.46	2.66**	.36
Non-randomness	3.08**	.41	2.58*	.25
One-value runs	4.08***	.51	3.36***	.44

Note. dfs = 72 for all contrasts.

*p<.05.

**p<.01.

***p<.001.

We compared the extent of non-randomness and the number of one-value runs across conditions. To assess non-randomness, we used the Wald-Wolfowitz test (also called the “runs test”), which determines the number of runs in a sequence that is expected if the entire sequence is random. For the present data, we calculated for each participant the absolute difference between the actual number of runs and the expected number of runs (if the participant’s responses were random)–higher scores indicate less randomness. Figure 1b presents the mean non-randomness in each condition; Table 1, middle line, presents the relevant contrasts. Both the correct-random and the incorrect-random conditions resulted in significantly greater deviations from random sequences (Ms = 4.64 and 4.20, respectively) than did the random-only condition (M = 1.90).

Next, we calculated for each participant the number of one-value runs for that participant’s “random” answers. Participants in the correct-random and incorrect-random conditions had significantly more one-value runs (Ms = 19.0 and 17.7, respectively) than did participants in the random-only condition (M = 11.6; see Figure 1c and Table 1, bottom line).

For all three variables of interest–correct response rate, non-randomness, and number of one-value runs–the differences between the correct- and incorrect random conditions were not significant, ts <1.

**Figure 2:**
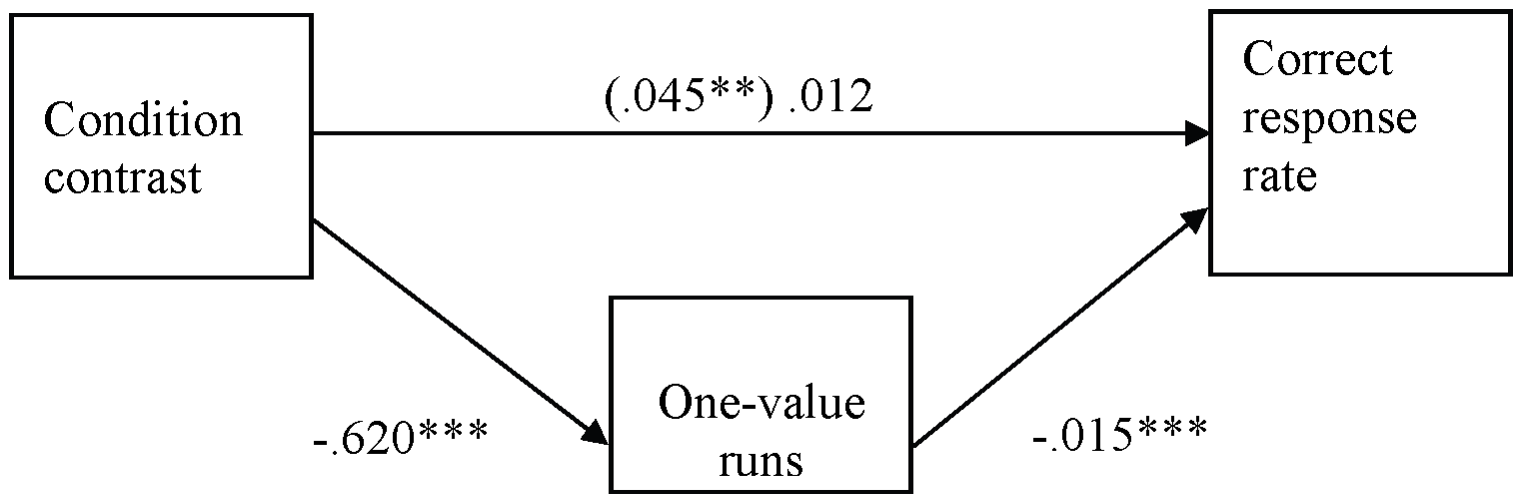
One-value runs as a mediator of the relation between condition contrast and correct response rate (Experiment 1). All coefficients are unstandardized. The coefficient in parenthesis does not control for the effect of one-value runs. **p<.01, ***p<.001.

We next examined whether the number of one-value runs mediated the effect of the experimental manipulation on the correct response rate. In this and the remaining studies, mediation was tested with the product-of-coefficients method [8]. In regression analyses, the experimental manipulation was operationalized by assigning −1 weight to the correct-random and incorrect-random conditions and +1 weight to the random-only condition. The mediation was significant (see Figure 2). The product of the path from the manipulation to the number of one-value-runs and the path from the number of one-value-runs to correct response rate was significant, z’ = 3.57, p<.01, and the relation between the experimental contrast and the correct response rate was rendered non-significant (p>.28) when the number of one-value runs was controlled for.

We also examined mediation by non-randomness but found it was less effective than mediation by one-value runs. The likely reason is that non-randomness is an omnibus measure, assessing whether the number of runs exceeds or falls short of the level expected by chance. The number of one-value runs, in contrast, represents the specific strategy that facilitated lowering the correct response rate.

The finding of lower correct response rate in both correct-random and incorrect-random conditions (relative to the random-only condition), mediated by higher number of one-value runs, supports the strategizing interpretation. However, the results also raise three issues that should be addressed.

First, alternating correct and incorrect answers is a simple strategy; why did participants in the random-only condition not use this strategy as well? The reason may have to do with how the questions and answers were structured. Correct answers were sometimes “yes” and sometimes “no.” Participants in the random-only condition who wished to alternate correct/incorrect answers would need to determine whether their answer (“yes” or “no) to the previous question was correct or incorrect, choose an opposite response in the current trial, and then decide whether “yes” or “no” represents the response they want to give. In contrast, participants in the correct-random and incorrect-random conditions needed only to determine whether their two responses (the baseline response and the “random” response) to the previous trial matched or mismatched (yes and yes as well as no and no would be matching responses; yes and no as well as no and yes will be mismatching responses). They would then choose to do the opposite in the current trial. Alternating between correct and incorrect responses was thus made easier for these participants than for the random-only participants.

Second, why did participants in the correct-random and incorrect-random conditions alternate correct and incorrect answers when the instructions warned them not to strategize? There may be at least two answers to this question. First, the instructions warned participants not to generate predictable patterns of yes/no responses but did not say anything about patterns of correct/incorrect responses. Furthermore, it has been established that in both perception and generation tasks, people miscalibrate randomness–they view binary sequences with more runs (or shorter runs) as more random unless the number of runs is excessive [6]. It follows that participants in the correct-random and incorrect-random conditions used a strategy that appeared to create a random sequences.

Finally, the proportion of correct responses in the incorrect-random condition was somewhat higher than the proportion of correct responses in the correct-random condition (this trend repeated itself in Experiment 3). Perhaps participants consume more cognitive resources when providing incorrect as opposed to correct responses, decreasing the resources available for strategizing–less strategizing would lead to higher correct response rate.

Although we believe the results of Experiment 1 support the strategizing interpretation, this interpretation is challenged by Sparrow and Wegner’s [1] Experiment 3. This study included correct-random and random-only conditions as well as two more conditions in which participants were asked to answer the questions randomly but were provided with either correct responses (correct-prime) or with incorrect responses (incorrect-prime) as the questions were read to them. The correct or incorrect responses appeared in the middle of the screen (without a title or explanation as to what they are); the “yes” and “no” options for the random choice appeared at the bottom of the screen.

On the surface, providing correct or incorrect responses should have facilitated strategizing and lower correct response rates as much as actual answering did. In other words, participants could have matched and mismatched their random responses with the correct (or incorrect) responses that were provided. However, neither the correct-prime nor the incorrect-prime lowered the correct response rate; in fact, the correct response rate in the incorrect-prime condition was higher than the corresponding rate in the random-only condition.

We believe that participants may be more likely to use their own answers (as opposed to responses provided by the computer) as a baseline when asked to generate random answers. In other words, when participants answer the same question twice, first correctly (or incorrectly) and then randomly, it is easy to alternate between matching and mismatching. It appears more difficult to do so when the first response does not come from the participant, particularly when the first response appears without any explanation about what it is and why it is there. More generally, participants may be more aware and more sensitive to responses that they themselves generated as opposed to responses that were given to them.

To test this interpretation, we replicated Sparrow and Wegner’s [1] Experiment 3 but changed the procedure somewhat. In particular, participants who received correct (or incorrect) responses were asked to copy them before answering randomly. We surmised that participants are more likely to “own” the responses they copy, facilitating the tendency to match and mismatch the random responses to what they copied. It was predicted, therefore, that participants copying the computer-provided responses would be able to lower their correct response rate, although perhaps not as much as participants who were asked to respond correctly. Given that participants copied (correct or incorrect) responses that were provided, the previous hypothesized difficulty in generating incorrect responses no longer applies. Therefore, it was expected that copying correct and copying incorrect responses would result in similar correct response rate. Finally, it was predicted that any reduction in the correct response rate in the two “copy” conditions would be due to strategizing and thus mediated by an increase in the number of one-value runs.

## Experiment 2

In this experiment, we re-ran the random-only and correct-random conditions from Experiment 1, and added copy-correct and copy-incorrect conditions. In the two copy conditions, participants copied the correct (or incorrect) responses provided by the computer before they answered the questions randomly.

### Methods

One hundred twenty-four undergraduates (86 females) took part in the study in exchange for course credit. They were assigned to one of four conditions. The procedure in the random-only and correct-only conditions was identical to that of Experiment 1.

In the two copy conditions, the participants were informed that following each question, the correct (or incorrect) answer would appear on the screen. Participants were instructed to copy the answer and then to answer randomly. In each trial, the question was displayed for 2 seconds before the answer appeared, and the answer (titled as correct or incorrect) was displayed for 2 seconds before the participant was asked to copy it. Once the participant copied the answer, the question appeared again on the next screen with the instruction to answer it randomly.

### Results and Discussion

**Figure 3:**
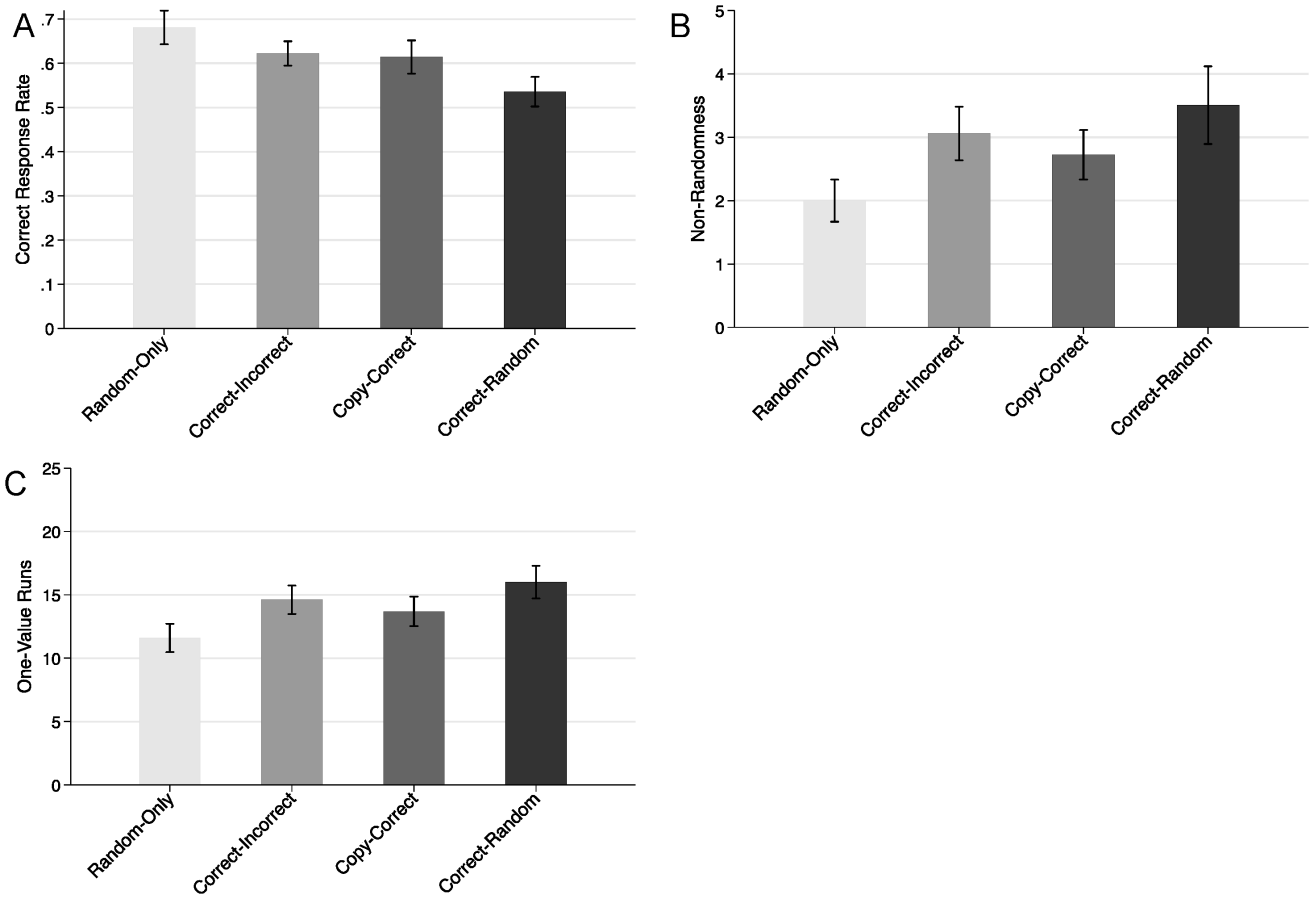
Means for (A) correct response rate, (B) non-randomness, and (C) one-value runs by experimental condition (Experiment 2). Error bars are standard errors of the means.

As in Experiment 1, the analyses examined three dependent variables–proportion of correct responses, non-randomness, and the number of one-value runs (see Figure 3 and Table 2).

**Table 2:** t-tests of Contrasts Among Conditions (Experiment 2).

Type of Contrast						
Dependent variable	Linear: correct-random (+1), copy conditions (0), random-only (−1)		Copy-correct and copy-incorrect vs. random-only		Copy-correct and copy-incorrect vs. correct-random	
	t	r	t	r	t	r
Proportion of correct responses	2.66**	.24	1.51†	.15	1.72††	.18
Non-randomness	2.20*	.20	1.70††	.17	1.04	.11
One-value runs	2.35*	.21	1.78††	.18	1.13	.12

Note. dfs = 120 for all contrasts.

† p = .13.

†† p<.10.

*p<.05.

**p<.01.

***p<.001.

Comparisons between the correct-random and random-only conditions replicated the results of Experiment 1. Participants in the correct-random condition (compared to the random-only condition) scored (1) lower on correct response rate, M = .54 vs. M = .68, (2) higher on non-randomness, M = 3.51 vs. M = 2.00, and (3) higher on one-value runs, M = 16.0 vs. M = 11.6, ps <.05 (see Figure 3).

For all three variables of interest, the copy-correct and copy-incorrect conditions were almost exactly midway between the correct-random and random-only conditions; for correct response rate Ms = .61 and.62, respectively; for non-randomness, Ms = 2.72 and 3.06, respectively; and for one-value runs, Ms = 13.7 and 14.6, respectively (see Figure 3). All differences between these two conditions were not significant, *t*s <1.

For all three variables, the linear contrast (random-only at one end, correct-random at the other end, and the two copy conditions in the middle) was significant (see left column in Table 2). For all three variables, the difference between the two copy conditions and either random-only or correct-random conditions sometimes approached significance but was never significant (see middle and left columns in Table 2).

**Figure 4:**
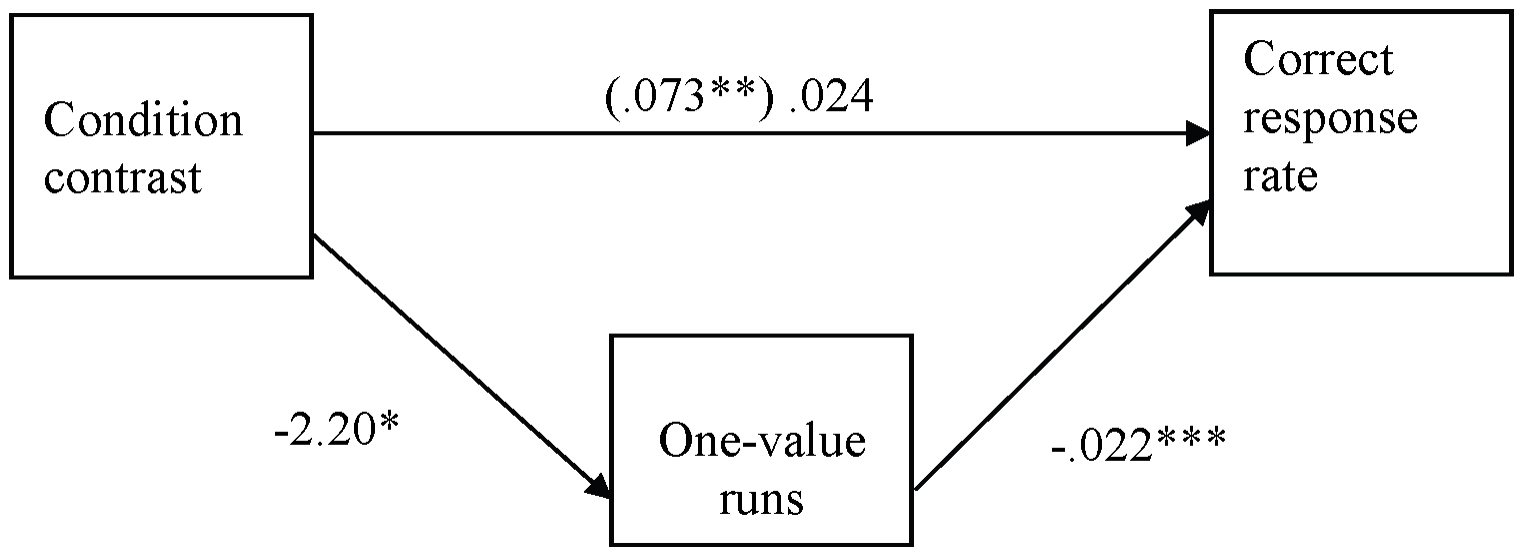
One-value runs as a mediator of the relation between condition contrast and correct response rate (Experiment 2). All coefficients are unstandardized. The coefficient in parenthesis does not control for the effect of one-value runs. **p<.01, ***p<.001.

We again examined whether the number of one-value runs mediated the relationship between experimental condition and proportion of correct responses (see Figure 4). In the regression analysis, the experimental manipulation was operationalized with −1 weight assigned to the correct-random condition, 0 weight assigned to the two copy conditions, and +1 weight assigned to the random only condition. The results showed significant mediation, z’ = 2.31, p<.05. The relationship between experimental conditions and proportion of correct responses was rendered non-significant (p>.18) when the number of one-value runs was controlled for.

Results were consistent with the hypothesis that requiring participants to copy answers promoted strategizing and thereby lowered correct response rates, though copying answers did not promote strategizing as effectively as requiring participants to answer questions themselves. As in Experiment 1, differences in correct response rates among experimental conditions appear to be a function of strategizing operationalized by the number of one-value runs.

## Experiment 3

Our core argument is that answering questions correctly or incorrectly (before answering them randomly) provides a base line, which allows participants to use a simple strategy to lower the correct response rate. An alternative interpretation is that answering the questions correctly (and perhaps also incorrectly) unprimes the tendency to answer correctly, thus resulting in lower correct response rate. Experiment 3 was designed as another, completely independent test of these two competing accounts. Our goal was to replicate the correct-random and the incorrect-random conditions in a way that allows strategizing but prevents unpriming. We predicted that allowing participants to strategize while preventing unpriming would lower the correct response rate and that the reduction would be mediated by an increase in the number of one-value runs.

Experiment 3 included the three conditions from Experiment 1 and added two more conditions: Paired correct-random and paired incorrect-random. In the paired conditions, participants answered correctly (or incorrectly) the odd numbered questions and randomly the even numbered questions. In the paired correct-random condition, they answered the first question correctly, the second randomly, the third correctly, and so on; in the paired incorrect-random condition, participants followed the same procedure except that they answered all odd numbered questions incorrectly rather than correctly. The correct and incorrect answers to the odd numbered questions could thus provide a baseline for the purportedly random answers to the even numbered questions without unpriming the tendency to answer the even numbered questions correctly.

Because of the hypothesized difficulty of generating incorrect responses, it was expected that the correct response rate in the incorrect-random condition would be somewhat higher than that of the correct-random condition. However, this difficulty is immaterial to the paired-incorrect condition. In this condition, the incorrect response and the random response were elicited by two different questions. The difficulty in answering a question incorrectly should not impact the ability to strategize when answering the next question randomly. Therefore, we expected that the correct response rates will be similar across the two paired conditions in the same way that it was similar across the two copy conditions in Experiment 2.

### Methods

Two hundreds seventy undergraduates (204 females) participated in the study for course credit. Fifteen additional undergraduates participated in the study but were excluded from the analyses because they had noted in the debriefing questionnaires that they had already done this study before. The participants were assigned to one of five conditions. The procedure in the random-only, correct-only, and incorrect-only conditions was identical to that of Experiment 1, except that the number of questions was increased to 80. In the two paired conditions, participants answered the odd numbered questions correctly (or incorrectly) and the even numbered questions randomly. Each question was accompanied by instructions to answer it correctly, incorrectly, or randomly.

### Results and Discussion

For three conditions (random-only, correct-random, and incorrect random), the variables of interest (proportion of correct responses, non-randomness, and the number of one-value runs) were calculated for the “random” responses to all 80 questions. For the two paired conditions, these variables were computed for the “random” responses to the 40 even-numbered questions; the non-randomness and one-value runs scores were then doubled.

**Figure 5:**
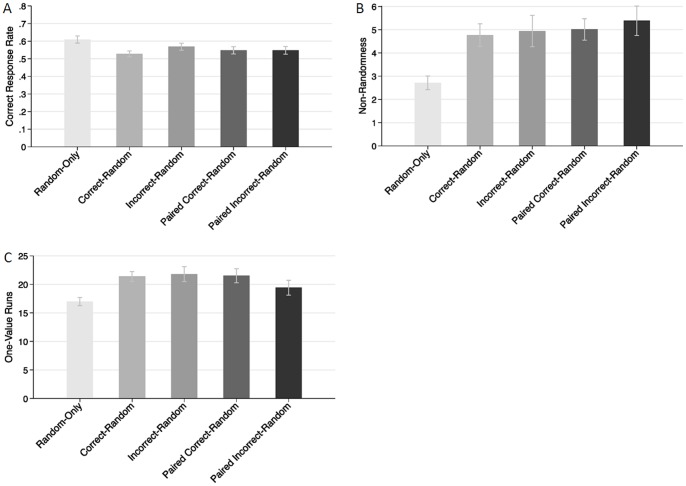
Means for (A) correct response rate, (B) non-randomness, and (C) one-value runs by experimental condition (Experiment 3). Error bars are standard errors of the means.

Figure 5 presents mean scores and standard errors for the three dependent variables by experimental conditions. We first tested our overall predictions with three contrasts. As expected, participants in the random-only condition (compared to participants in all other conditions) scored significantly higher on correct response rate and significantly lower on both non-randomness and the number of one-value runs (see left column in Table 3).

**Table 3:** t-tests of Contrasts Among Conditions (Experiment 3).

Type of Contrast						
	Random-only vs. all other conditions		Incorrect and correct-random vs. random-only		Paired conditions vs. random-only	
	t	r	t	r	t	r
Proportion of correctresponses	2.84**	.17	2.56*	.19	2.56*	.20
Non-randomness	4.20***	.25	3.51***	.26	4.08***	.31
One-value runs	3.51***	.21	3.60***	.27	2.72**	.21

Note. dfs = 265 for all contrasts.

*p<.05.

**p<.01.

***p<.001.

We followed with three sets of orthogonal contrasts that were not expected to yield significant differences. The first set showed that incorrect-random and correct-random conditions did not differ on any of the three dependent variables, *t*s <1.39; the second set showed that the paired random-incorrect and the paired random-correct also did not differ on any dependent variable, *t*s <1; the third set showed that the incorrect-random and correct-random conditions did not differ from the corresponding paired conditions on any dependent variable, *t*s <1.03.

Because the rationale for predicting lower correct response rate in the incorrect-random and correct-random conditions was somewhat different from the rationale for predicting lower correct response rate in the two paired conditions, it was advisable to compare each of these 2-condition groups separately to the random-only condition. These comparisons showed that participants in the random-only condition scored significantly higher on correct response rate, M = .61, (1) compared to participants in the incorrect-random and correct-random conditions (Ms = .57 and.53, respectively), and (2) compared to participants in the paired incorrect-random and paired correct-random conditions (Ms = .55 and.55; see middle and right columns in Table 3). Participants in the random-only condition scored significantly lower on non-randomness, M = 2.71, (1) compared to participants in the incorrect-random and correct-random conditions (Ms = 4.94 and 4.77, respectively), and (2) compared to participants in the paired incorrect-random and paired correct-random conditions (Ms = 5.39 and 5.01, respectively). Finally, participants in the random-only condition scored significantly lower on the number of one-value runs, M = 17.0, (1) compared to participants in the incorrect-random and correct-random conditions (Ms = 21.8 and 21.4, respectively), and (2) compared to participants in the paired incorrect-random and paired correct-random conditions (Ms = 19.4 and 21.5, respectively).

As in Experiment 1, the correct response rate in the incorrect-random condition (M = .57) was somewhat higher than the corresponding rate in the correct-random condition (M = .53; *t* = 1.38) and, in fact, was only somewhat lower than the rate in the random-only condition (M = .61; *t* = 1.37). However, when results for these three conditions from Experiments 1 and 3 are combined, the correct rate in the incorrect-random condition is significantly lower than that of the random-only condition, Z = 3.36, p<.001, but not significantly higher than that of the correct-random condition, Z = 1.42, p>.15.

**Figure 6:**
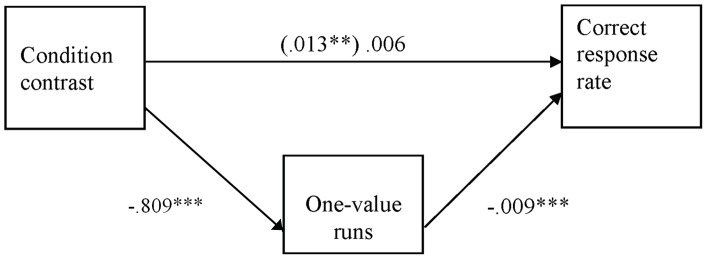
One-value runs as a mediator between condition contrast (random-only vs. all other conditions) and correct response rate (Experiment 3). All coefficients are unstandardized. The coefficient in parenthesis does not control for the effect of one-value runs. ***p<.001.

Next, we examined whether the number of one-value runs mediated the difference between the random-only condition and all other conditions in correct response rate (see Figure 6). The results showed a significant mediation, z’ = 3.28, p<.01. The significant relation between the contrast and the correct response rate was rendered non-significant (p>.13) when the number of one-value runs was controlled for.

Experiment 3 showed that it is not only the incorrect-random condition that lowered the correct-response rate; the two paired condition did so as well. Unpriming cannot account for the difference observed between the paired conditioned and the random-only condition. Furthermore, as predicted by a strategizing explanation, the higher correct response rate in the random-only condition, compared to all other conditions, was mediated by an increase in the number of one-value runs.

## General Discussion

Sparrow and Wegner [1] suggested that answering questions correctly induces unpriming, i.e., it eliminates the influence of knowledge of the correct answer, leading to random responding and correct response rates that are close to chance. An alternative explanation is that the lower correct response rate is due to strategizing, not unpriming. According to the strategizing account, answering correctly serves as a baseline, allowing participants to alternate between matching and mismatching their correct responses. This strategy lowers the correct response rate but, contrary to Sparrow and Wegner’s [1] predictions, also leads to less random responses.

The evidence in support of the strategizing explanation is based on results obtained in two different sets of circumstances–the incorrect-random condition and the paired conditions–that for different reasons both allowed strategizing but not unpriming. Both sets of circumstances yielded lower correct response rates when compared to a random-only condition. As noted earlier, however, our explanation of the results in the incorrect-random condition can be challenged on the grounds that answering questions incorrectly makes people think about the correct responses, leading to unpriming.

This argument, however, does not stand the test of logic and is contradicted by research findings. Priming increases construct accessibility and the higher accessibility may be translated to behavior. As Sparrow and Wegner [1, p. 1009] stated, “when an experience primes a person to think about something, the person’s behavior and judgment may be influenced by the prime.” But if thinking alone results in unpriming, all priming automatically leads to unpriming without any behavioral consequences. Obviously, this does not happen. For example, although participants in the random-only condition must have thought about the correct answer before they answered “randomly,” they certainly were not unprimed. In a similar way, thinking about the correct response before answering them incorrectly also cannot unprime. More generally, our argument is that behavior may unprime; cognition about behavior may not.

Sparrow and Wegner [1] actually tested directly the notion that cognition alone unprimes in their aforementioned Experiment 3. In this study, participants were exposed to correct (or incorrect) answers in order “…to determine whether mere knowledge activation might be effective in unpriming” (p. 1013). Participants exposed to this knowledge provided at least as many correct answers when asked to answer randomly as participants in the random-only condition. According to Sparrow and Wegner [1, p. 1014), this result “… suggests that the mere thought of the correct response is not sufficient to initiate unpriming and that the actual response may be required to produce an unpriming effect”.

Sparrow and Wegner’s [1] Experiment 4 was another, albeit indirect test of whether thought alone can unprime. They had one group of participants tap a computer key under the desk to indicate the correct response before answering the same question randomly (mere expression condition), and another group indicate by tapping a computer key whether or not they knew the answer before answering randomly (self-presentation condition). Tapping a correct response provides a baseline while indicating whether one knows the answer does not. Indeed, Sparrow and Wegner [1] found that the mere expression condition lowered the correct response rate (relative to a random-only condition) while the self-presentation condition did not. Participants in the self-presentation condition must have thought about the correct answer before indicating whether they knew it, again indicating that thinking alone does not unprime.

In any event, the paired conditions in Experiment 3 provided independent evidence that it was strategizing, rather than unpriming, that led to lower correct response rates in the correct-random condition. In Experiment 3, participants answered odd numbered questions correctly (or incorrectly), allowing them to strategize and thus obtain a lower correct response rates when they answered the even numbered questions randomly. Unpriming cannot explain this result because answering a question correctly or incorrectly, does not uprime the tendency to answer the next, distinct question correctly. Strategizing is thus the most parsimonious explanation for results obtained in four different conditions (incorrect-random, correct-random, and the two paired conditions), all of which showed lower correct response rates relative to the random-only condition.

Furthermore, the lower correct response rates in the four conditions were accompanied by less randomness, and were mediated by higher numbers of one-value runs. According to Sparrow and Wegner [1], unpriming was supposed to increase rather than decrease randomness. Indeed, if unpriming removes a tendency to answer questions correctly, then no strategy is necessary to achieve close to.5 correct response rate. Our results, however, indicate that strategy was the mechanism that accounts for the lower correct response rate.

It is important to note that our critique is only aimed at the particular paradigm that Sparrow and Wegner [1] used to test the unpriming model, but not at the model itself. Stated differently, the idea that enactment of primed thoughts leads to deactivation of the prime remains viable, but it is yet to be empirically tested. We recognize that the reader may wonder if the present work accomplished anything besides providing an alternative interpretation of Sparrow and Wegner’s [1] results. Actually, we believe that one of our findings may prove interesting to research on the ability to judge and generate random binary events.

Consistent with previous research [6], our participants systematically failed to generate true random sequences of binary events. They also displayed a bias that, to the best of our knowledge, was not noted in previous research. In constructing too short runs (the well-known gambler’s fallacy), they relied mostly on events (answers to the same or previous question) that they generated and tended to ignore events generated by the computer. Future research may examine whether this tendency generalizes to contexts other than the present paradigm.
